# SDBS-AEO Mixture for Triton X-100 Replacement: Surface Activity and Application in Biosensors

**DOI:** 10.3390/bios14100505

**Published:** 2024-10-16

**Authors:** Zhenzhen Li, Lei Wang, Mengjie Tang, Yulong Sun, Li Zhang, Zhongxiu Chen

**Affiliations:** 1 Molecular Food Science Laboratory, College of Food & Biology Engineering, Zhejiang Gongshang University, Hangzhou 310018, China; lizhenzhen199808@163.com (Z.L.); zheligong3wl@163.com (L.W.); tangmj1998@163.com (M.T.);; 2 Acon Biotech (Hangzhou) Co., Ltd., Hangzhou 310030, China

**Keywords:** TX-100 replacement, surfactants, synergistic interaction, biosensors

## Abstract

Triton X-100 (TX-100) is a commonly used surfactant in the manufacture of biosensors. The factors limiting the use of TX-100 in biosensors are environmental concerns. In this study, the binary system of sodium dodecyl benzene sulfonate (SDBS) and fatty alcohol-polyoxyethlene ether (AEO) was investigated from the physicochemical principle of surfactant interaction and its application in biosensors. The results demonstrated that a mixture of SDBS and AEO at an appropriate molar ratio had a comparable activity to TX-100 in terms of surface activity, micelle formation, dynamic adsorption, foaming, emulsifying, and cell permeability. Theory and experimentation support the idea that SDBS-AEO might take the place of TX-100 in the manufacturing of biosensors. This study contributes to the development of alternatives to TX-100 and provides a new perspective for an in-depth study of the interaction mechanism of additives in biosensor design.

## 1. Introduction

The surfactant Triton X-100 (TX-100) exhibits excellent surface activity, emulsification, and cell permeability compared to conventional surfactants [1]. It has wide-ranging applications in the nanomaterials preparation, drug delivery, and biosensors industry [2,3,4]. However, it has been noted that TX-100 degradation products such as nonylphenol (NP) have potentially harmful effects on the environment and human health, showing severe toxicity to plankton, amphibians, invertebrates, and fish [5]. Additionally, NP interacted with nuclear hormone receptors, including those for estrogen, androgen, and progesterone, leading to multiple toxic effects both in vivo and in vitro [6]. China prohibits the use of nonylphenol to produce nonylphenol polyoxyethylene ether, and the European Union and USA have restricted their use by their inclusion in the list of priority hazardous substances for surface waters [7].

As a result of environmental concerns regarding ecotoxic byproducts and the biosensor export business, there is an urgent need to discover functionally equivalent surfactants that might replace TX-100 in biosensor design. As an eco-friendly substitute for TX-100, researchers have been searching for surfactants such as nonylphenol ethoxylate carboxylate (NCEO), fatty alcohol-polyoxyethlene ether (AEO), “Nereid” (a newphenol-free detergent), and alkyl glucoside (APG) [8,9,10,11]. However, it is a challenge to find an alternative detergent to eventually replace TX-100. For example, AEO has excellent emulsifying, wetting, and dispersion qualities, but its total effectiveness is inferior to that of TX-100. APG is prone to hydrolysis under acidic conditions, which limits its application. TX-100 could disturb the lipid bilayer and essential membrane proteins, which affected the cell structure and ultimately resulted in cell lysis [12] in biosensor manufacturing. “Nereid” had comparable virus inactivation abilities to TX-100 and efficiently deactivated viruses in plasma when treated with solvent–detergent at a standard temperature [9]. Tan et al. [13] demonstrated that the surfactant “Simulsol SL-11W” (non-ionic non-ethoxylated surfactant prepared from glucose and C11 fatty alcohol) induced only reversible alterations in membrane morphology, while TX-100 caused complete and irreversible membrane solubilization. Lim et al. [14] synthesized a polyethylene glycol ether surfactant using mannitol and caprylic acid, which exhibited excellent adhesive properties and could be used as a TX-100 replacement in emulsion polymerization.

This study focuses on formulation using commercially available surfactants. Following the desire for minimal differences between TX-100 and the potential replacement candidates, non-ionic fatty alcohol-polyoxyethlene ether (AEO) was selected because it is a polyethylene glycol-based detergent with a low critical micelle concentration (CMC). Anionic sodium dodecyl benzene sulfonate (SDBS) was selected as another component because it is different from TX-100 and does not contain the nonylphenol moiety. Moreover, the phenol group of SDBS might give the surfactant excellent membrane permeability. The ready biodegradability of the components or their degradation to nontoxic metabolites are the desirable features. The synergistic interaction of the surface activity, critical micelle concentration, hemolytic activity, foaming, and emulsifying properties of the SDBS-AEO binary mixture was investigated, with a focus on evaluating the potential of the SDBS-AEO complex system as a substitute for TX-100. Furthermore, the application of SDBS-AEO surfactant in biosensors was explored and compared with TX-100. This research is of significant importance for the development of environment-friendly surfactants and alternatives to TX-100 and provides a basic science for biosensor design.

## 2. Materials and Methods

### 2.1. Materials

Triton X-100 (TX-100, CAS 9036-19-5), sodium dodecyl benzene sulfonate (SDBS, CAS 25155-30-0), palmitic acid (CAS 57-10-3, purity > 98%), p-nitrophenol (p-NP, CAS 100-02-7, purity > 99%, 4-Nitrophenol), and bile salts (NaC, purity > 97%, CAS 361-09-1, Sodium cholate) were purchased from Sigma-Aldrich (Shanghai, China) and were of analytical grade. Fatty alcohol-polyoxyethylene ether (AEO, CAS 68131-39-5), corn oil (CAS 8001-30-7), liquid paraffin (CAS 8042-47-5), and p-nitrobenzoic acid ester (p-NPB, CAS 2487-26-5, purity ≥ 98%, 4-Nitrophenyl butyrate) were purchased from Aladdin Shanghai Co., Ltd. (Shanghai, China). Rabbit red blood cells (stored in 2% PBS, Catalog No. S33570) were purchased from Yuanye Shanghai Co., Ltd. (Shanghai, China). Ultrapure water (18.25 MΩ·cm) was used in all the experiments, and it was purified by a Nanopure water purification system (Millipore) from Hangzhou Yongqieda Purification Technology Co., Ltd. (Hangzhou, China).

### 2.2. Sample Preparation

A stock solution (2 mmol/L) of TX-100, AEO, and SDBS was prepared by dispersing the compounds in ultrapure water and stirring for at least 3 h. SDBS and AEO at varying molar ratios were mixed for the determination of critical micelle concentration, foaming abilities, wetting abilities, and other application characteristics.

### 2.3. Determination of the Surface Tension

Surface tension was obtained using a tensiometer (Kruss-K100, Hamburg, Germany). The surface tension was measured by using the hanging drop method, which measured the surface tension of the droplet according to its shape. The surfactant solution was prepared with a concentration range of 0.001–0.00001 mol/L at 25 °C. The apparent surface tension of each sample was measured 5 times within a 2 min interval between each reading.

### 2.4. Micellization and Synergistic Parameters of Binary Surfactant Mixtures

The theoretical calculation of surface properties, micelle formation, synergistic effects, and thermodynamic behavior of the surfactant mixtures referred to the literature [15,16]. The detailed calculations are as follows:

(1) $${pc}_{20}=-lgc_{20}$$

(2) $$\Gamma_{max}=-\frac{1}{RT}{\left(\frac{d\gamma }{dlog\;c}\right)}_{T}=-\frac{1}{2.303RT}{\left(\frac{d\gamma }{dlog\;c}\right)}_{T}$$

(3) $$A_{min}=\frac{1}{N_{A}*\Gamma_{max}}$$

(4) $$\beta^{M}=\frac{ln(\frac{\alpha_{1}CMC}{X_{1}{CMC}_{1}})}{{\left(1-X_{1}\right)}^{2}}$$

(5) $$\beta^{S}=\frac{ln(\frac{\alpha_{1}c_{12}^{0}}{X_{1}^{S}C_{2}^{0}})}{{\left(1-X_{1}^{S}\right)}^{2}}\;\;\;\;\;\;\;\;\;\;\;\;\;\;$$

In the equation, *p*$c_{20}$ represents the concentration of the surfactant to reduce the surface tension by 20 mN/m, $\Gamma_{max}$ represents the molar concentration at which the adsorption of the surfactant reaches saturation, and $A_{min}\;$ represents the minimum area per molecule at maximum interfacial coverage. *R* is the gas constant 8.314 J/(mol·K), T is the absolute temperature (298.15 K), and $N_{A}$ is the Avogadro’s constant 6.022 × 10^23^/mol. ${\;\beta }^{M}$ represents the parameter of the interaction between surfactants in mixed micelles, reflecting the deviation of mixed micelles from ideal mixing.

$X_{1}$ was calculated by the following formula using a trial-and-error method:

(6) $$\frac{X_{1}^{2}ln(\frac{\alpha_{1}CMC}{X_{1}{CMC}_{1}})}{{\left(1-X_{1}\right)}^{2}ln\left[\frac{(1-\alpha_{1})CMC}{(1-X_{1}){\alpha_{1}CMC}_{2}}\right]}=1$$

The hybrid adsorption layer composition ($X_{1}^{S}$) and interaction parameters ($\beta^{S}$) were derived from the following equations:

(7) $$\frac{{X_{1}^{S}}^{2}ln(\frac{\alpha_{1}c_{12}^{0}}{X_{1}^{S}c_{1}^{0}})}{{\left(1-X_{1}\right)}^{2}ln\left[\frac{(1-\alpha_{1})c_{12}^{0}}{(1-X_{1}^{S})c_{2}^{0}}\right]}=1$$

In the equation, $X_{1}^{S}$ represents the SDBS in the mixed monolayer. $c_{1}^{0}$, $c_{2}^{0}$, and $c_{12}^{0}$ are the concentrations (mmol/L) of SDBS, AEOn, and the SDBS-AEOn complex at a surface tension of 52 mN/m, respectively.

### 2.5. Thermodynamics of Mixed Micelles of the Binary Surfactant Mixtures

The hydrophobic interactions of surfactants in aqueous solution include two aspects: firstly, the adsorption of surfactant molecules at the air–liquid interface, where the surfactants diffuse from the bulk phase to the interface, forming an oriented adsorption layer; secondly, the formation of micelles in the bulk phase, where the alkyl chains aggregate, thereby distancing themselves from water molecules [17]. To fully understand the spontaneous formation of mixed micelles, the thermodynamics and mixed adsorption of the SDBS-AEOn system were further investigated. The thermodynamic functions of the SDBS-AEOn mixed system were formulated using the regular solution theory as follows (Equations (8)–(10)) [16]:

(8) $${\Delta H}^{M}=RT(X_{1}lnf_{1}+X_{2}lnf_{2})$$

(9) $${\Delta G}^{M}=RT(X_{1}lnf_{1}X_{1}+X_{2}lnf_{2}X_{2})$$

(10) $${\Delta S}^{M}=\frac{{\Delta H}^{M}-{\Delta G}^{M}}{T}$$

In the equation, ${\Delta H}^{M}$ represents the enthalpy change of the mixture, ${\Delta G}^{M}$ represents the experimentally obtained free energy change of the mixture, ${\Delta G}_{ideal}^{M}$ represents the theoretically obtained free energy of the mixture, ${\Delta S}^{M}$ represents the entropy change of the mixture, R is the gas constant (8.314 J/(mol·K)), and *T* is the absolute temperature (298.15 K).

### 2.6. Dynamic Adsorption Parameters of Binary Surfactant Mixstures

The surface tension of the SDBS-AEOn system at 25 °C was determined by the pendant drop method. Various parameters were calculated, and the adsorption mechanism was studied.

In the case of c < CMC, following Miller’s theory [18], the modified Ward–Tordai equation was used as follows:

(11) $$\mathrm{Initial}\;\mathrm{stage}\;\mathrm{of}\;\mathrm{adsorption}:\;{\gamma (t)}_{t\to 0}=\gamma_{0}-2nRTC{\left(\frac{D_{a}t}{\pi }\right)}^{\frac{1}{2}}$$

(12) $$\mathrm{Late}\;\mathrm{stage}\;\mathrm{of}\;\mathrm{adsorption}:\;{\gamma (t)}_{t\to \infty }=\gamma_{eq}-\frac{nRT\Gamma^{2}}{C}{\left(\frac{\pi }{{4D}_{a}t}\right)}^{\frac{1}{2}}$$

In the equation, $D_{a}$ is the apparent diffusion coefficient (m^2^/s), R is the gas constant (8.314 J/mol·K), T is the temperature (K), Γ is the surface adsorption capacity (mol/m^2^), and c is the surfactant concentration (mol/L).

In the case of c > CMC, the Gibbs equation and Miller asymptotic equation were used:

(13) $$\gamma =\gamma_{eq}+\frac{RT\Gamma^{2}}{C_{0}t}{\left(\frac{1}{Dk}\right)}^{\frac{1}{2}}$$

(14) $$\frac{\frac{d\gamma }{dt^{\frac{1}{2}}}(C=CMC)}{\frac{d\gamma }{dt^{-1}}(C>CMC)}={\left(\frac{k\pi }{4}\right)}^{\frac{1}{2}}$$

In the equation, the micelle dissociation constant *k* can be obtained by plotting γ against t^−1^.

### 2.7. Determination of Interfacial Tension

Interfacial tension was measured using an automated drop tensiometer (Kruss, DVT-50, Hamburg, Germany) according to the Du Noüy method. The dynamic interfacial tension of the surfactant–model oil interface was investigated using the pendant drop technique. Samples with 2 mmol/L and a variable ratio were measured to obtain their dynamic interfacial tensions.

### 2.8. Determination of Wettability

The contact angle can intuitively reflect the wetting performance of surfactant solutions on solid surfaces. The contact angles of several surfactant solutions on the lipophilic surface were measured using a contact angle instrument (Biolin Scientific, Gothenburg, Sweden) at 25 °C. The droplet sizes were all set to 6 μL. Five measurements were performed and averaged.

### 2.9. Determination of Foaming Properties

An amount of 20 mL of surfactant solution with a concentration of 0.5 g/L was prepared and then transferred to a 100 mL stoppered cylinder. The resulting mixture was vigorously shaken 50 times, and the foam heights at 0 and 5 min were recorded. The above operation was repeated five times, and the average value was taken as a measure of the foaming properties of the surfactant [19].

### 2.10. Determination of Emulsification Properties

An amount of 0.2 g of surfactant compounds was dissolved in 20 mL of ultrapure water, and then 20 mL of the solution and 20 mL of oil was transferred into a 100 mL stoppered cylinder. The obtained mixture was allowed to settle for 1 min after shaking it up and down vigorously 10 times, and the above operation was repeated 5 times. The time used to separate 10 mL of water from the oil–aqueous emulsion was taken for an evaluation of the emulsion’s stability [20].

### 2.11. Determination of Cell Membrane Permeability

Rabbit red blood cells kept in PBS buffer were used to investigate the hemolytic activity of surfactants. A standard protocol involves combining 200 μL of red blood cell suspension with 800 μL of surfactant at varying concentrations (ranging from 0.1 CMC to 20 CMC) in a 1.5 mL centrifuge tube. After incubating for 30 min at 25 °C, the mixture was centrifuged at 3000 rpm for 10 min. The absorbance of the supernatant was measured at a wavelength of 540 nm. The formula for calculating the hemolysis rate can be found in reference [21].

### 2.12. Evaluation of SDBS-AEO Mixture for Triton X-100 Replacement in Biosensors

SDBS-AEO mixture was used instead of TX-100 to prepare the raw stock solution for glucose sensors. An amount of 1–3 μL solution was dispensed in the electrode area of the test strip (covering the working electrode and counter electrode), and dried for 5–10 min to prepare the test strip. The prepared reagent strips were used on the electrochemical workstation or the commercial meter to detect a series of sample solutions containing glucose. Using the chronoamperometry method, the change in current over time was recorded to obtain a current–time curve. The current value at 5 s was selected, and each sample was tested 5 times to obtain the 5 s current value at different concentrations. The linear graph, current average, (Standard Deviation) (SD) and Coefficient of Variation (CV) values were then analyzed.

### 2.13. Data Statistical Analysis

All measurements were conducted in at least three independent experiments. Origin Pro 8.0 was used for plotting and data processing. The single-factor Duncan analysis method using SPSS 23.0 statistical software was used for significance difference testing.

## 3. Results and Discussion

### 3.1. Screening of Surfactant Monomers

Surfactant mixtures often exhibit synergistic effects, which outperform single surfactants in terms of application performance. The synergistic effect is due to the non-ideal mixing within the aggregates, which reduces the critical micellar concentration and interfacial tension, thereby enhancing the overall performance of the mixture compared to the surfactant monomers. In order to obtain the best synergistic effect, surfactant monomers need to be screened.

Table 1 lists the surface-active parameters of 10 commonly used anionic and non-ionic surfactants. Among the ionic surfactants (SDBS, SLS, NLSS, SDS, SBS, and AES), SDBS has the lowest CMC; this may be attributed to its smaller repulsion between hydrophobic chains. The maximum saturated adsorption capacity (Γ_max_) and the minimum molecular cross-sectional area (A_min_) were primarily determined by molecular structure and chain length. A higher Γ_max_ and lower A_min_ indicate greater efficiency in reducing surface tension. Table 1 shows that the Γ_max_ and A_min_ of AEO are close to TX-100. This is possibly because the high flexibility in the polyethylene oxide ether chain can reduce the intermolecular steric hindrance, which increases the adsorption amount and decreases adsorption area. A higher pC_20_ indicates that a surfactant can reduce surface tension at the air–water interface at lower concentrations more effectively, which is a measure of its adsorption efficiency at the surface. Compared to TX-100, SDBS has the highest adsorption efficiency possibly due to its benzene ring structure, which enhances hydrophobicity and promotes gas–liquid interface adsorption [22].

### 3.2. Determination of the Optimal Ratio of the SDBS-AEOn Mixture

#### 3.2.1. Theoretical Calculation of the Synergistic Interaction Parameters of SDBS-AEOn Mixtures

The above results show that the ionic surfactant SDBS and non-ionic surfactant AEO exhibited significant capabilities in reducing surface tension and could be good monomers for acting as substitution candidates of TX-100. Therefore, in the following study, binary mixtures of SDBS with AEO with different EO numbers (*n* = 3, 7, 9) at different mole fractions were investigated.

Table 2 shows that as the number of EO units increases, the hydrophilicity of AEOn improved, leading to a higher minimum surface tension. Compared to TX-100, the mixture of SDBS and AEOn exhibited a more significant reduction in surface tension, with the SDBS-AEO-9 (1:9) mixture performing similarly to TX-100. The CMC increased and γ_CMC_ decreased as the SDBS ratio increased, particularly in the SDBS-AEO-9 system. This result may be because the EO chains of AEO-9 covered the charged headgroups of SDBS, reducing charge repulsion and lowering surface charge density, which makes it easier for SDBS to enter the mixed micelles [23].

In order to gain further insights into the interaction between the constituent surfactants in the mixed micelle and to determine the mixed micelle composition, the interaction parameter ${(\beta }^{M})$ was evaluated using the regular solution theory developed by Rubingh and Holland [24]. As shown in Table 2, the $\beta^{M}$ value in the SDBS-AEOn mixed micelles was negative, with the values $\left|\beta^{M}\right|$ > ln$\left|\frac{{CMC}_{1}}{{CMC}_{2}}\right|$. These results indicated that the SDBS-AEOn mixed micelles had a mutual attraction and showed a positive synergistic effect. In the SDBS-AEOn system (*n* = 3, 9), the absolute value of $\beta^{S}$ was greater than ln$\left|\frac{c_{1}^{0}}{c_{2}^{0}}\right|$, suggesting enhanced efficiency in reducing surface tension. As the EO chains in AEOn molecules increased, the interaction parameter ($\beta^{M}$) value decreased, indicating that the mutual attraction in the mixed micelles became stronger with shorter EO chains. In the SDBS-AEO-9 system, $\left|\beta^{M}\right|$ was the largest at $\alpha_{1}$ = 0.1, indicating the strongest synergistic effect. Across all systems, $\left|\beta^{S}\right|$ was the highest in the case of SDBS-AEO-9 (1:9), suggesting that the positive synergistic effect between SDBS and AEO-9 was most pronounced in the mixed adsorption layer at this ratio. Thus, the SDBS-AEOn (*n* = 9) mixed system found it easier to form micelles in the solution and was more closely arranged in the adsorption layer on the surface of the solution.

The- thermodynamic parameters of the mixed micelles of the SDBS-AEOn mixtures were further investigated. Table 2 shows that the ΔG^M^ were all negative, indicating spontaneous micelle formation. Negative ΔH^M^ values suggest exothermic micellization, while positive ΔS^M^ values highlight entropy-driven micelle formation. The hydrophobic effect (TΔS^M^ > ΔH^M^) was the main force driving micelle aggregation. The results showed that the micellization process of the SDBS-AEO hybrid system was driven by both entropy and enthalpy. In the SDBS-AEOn (*n* = 9) system, the mixed micelle achieved its highest stability when $\alpha_{1}$ = 0.1.

#### 3.2.2. Calculation of the Dynamic Adsorption Parameters of SDBS-AEOn Mixtures

Table 3 lists the apparent diffusion coefficients ($D_{a}$) and micelle dissociation constants (*k*) of SDBS-AEOn mixtures at various concentrations. As the number of EO units increased, the adsorption rate decreased, while the $D_{a}$ value increased. AEO-9 with a longer chain had a maximum value, which may be the result of the diffusion and hydrophobicity of surfactant molecules competing with each other in the bulk phase. Paweena et al. [25] also found that the structure of both the hydrophobic and hydrophilic parts significantly impacts adsorption, with a larger hydrophobic component leading to a higher adsorption potential barrier and more difficult adsorption.

In the initial stage of adsorption, the *D_a_* value of the SDBS-AEOn system was between the values of a single surfactant, and changed with the different proportions of SDBS, indicating that the composition of the SDBS-AEOn compound system was an important factor affecting the surface adsorption. The Da in the late stage of adsorption was much smaller than that in the early stage of adsorption, indicating that the adsorption process of the SDBS-AEO compound system was in line with the mixed–controlled adsorption mechanism. The apparent diffusion coefficient of SDBS-AEO-9 (1:9) reached its highest value of 5.93 m^2^/s. The reason is likely related to the increased molecular mobility of EO units, which accelerated the surfactant diffusion and rapidly decreased the surface tension of the aqueous solution.

The smaller the *k*, the more stable the micelles. The values of *k* of the AEO-SDBS mixture were between the single surfactants, indicating that the AEO and SDBS stabilized the micelles to a certain extent. When α = 0.1, the minimum *k* value of the SDBS-AEOn (*n* = 9) compound system was 1.31 s^−1^, and the micelles were in the most stable state.

#### 3.2.3. Dynamic Interface Tension

**Figure 1:**
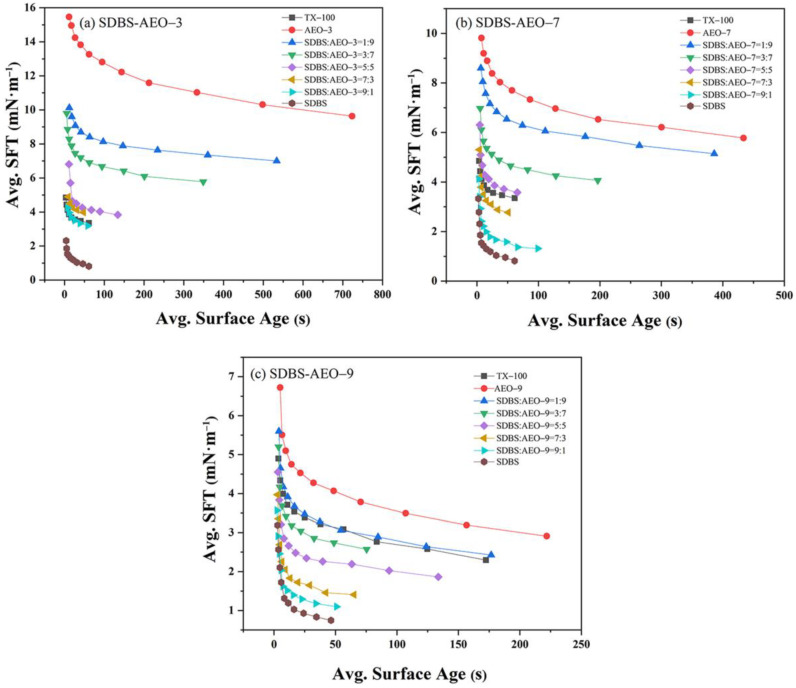
Dynamic interfacial tension curves of SDBS-AEOn (2 mmol/L) complex system. (**a**) SDBS-AEO-3, (**b**) SDBS-AEO-7, (**c**) SDBS-AEO-9.

The macroscopic surface activity of the surfactants is related to the microscopic surface tension and interfacial tension. The above hybrid micelle properties, synergistic interaction, and dynamic adsorption parameters are all related to surface tension. Moreover, the interfacial tension is also a critical factor in evaluating the performance of mixed surfactants at the liquid–liquid interface. Figure 1 illustrates the dynamic interfacial tension of the SDBS-AEOn system. The interfacial tension of the composite system was influenced by the number of hydrophilic chains (EO chains). A higher number of EO groups led to a greater reduction in interfacial free energy, resulting in a lower interfacial tension for AEO-9. Research has shown that more polyethylene oxide (EO) or polypropylene oxide units enhanced the ability to form a stable interface [26]. Surfactants with longer EO chains created a more ordered and thicker monolayer at the interface, significantly lowering interfacial tension [27]. Increasing the SDBS content resulted in an interfacial tension closer to that of single SDBS. The SDBS-AEO-9 (9:1) mixture achieved an interfacial tension of only 1.10 mN/m. Additionally, the trend in interfacial tension for SDBS-AEO-9 (1:9) closely resembled that of TX-100, indicating that SDBS and AEOn synergistically were adsorbed at the oil/water interface, with SDBS having a more significant impact on reducing interfacial tension.

### 3.3. Macroscopic Properties of SDBS-AEOn Mixtrure

#### 3.3.1. Wettability

**Figure 2:**
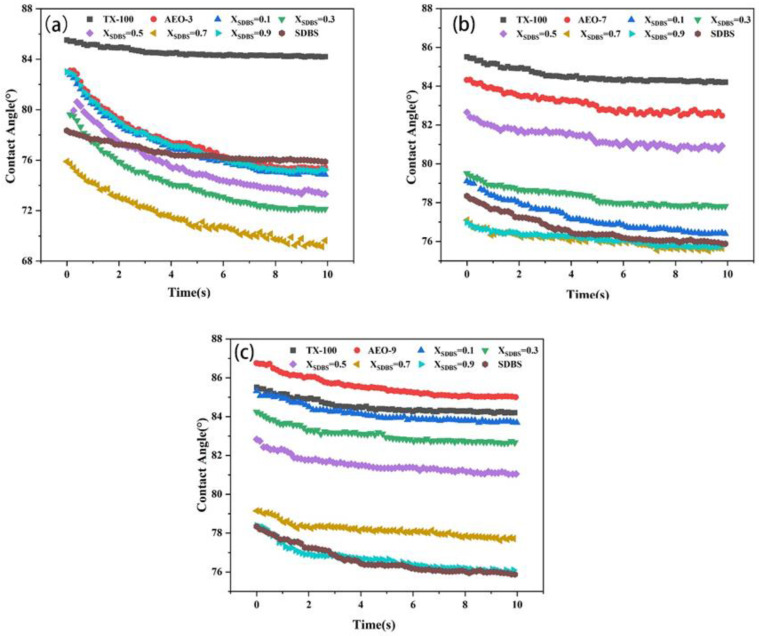
Dynamic contact angle change curves of SDBS-AEOn (2 mmol/L) system. (**a**) SDBS-AEO-3, (**b**) SDBS-AEO-7, (**c**) SDBS-AEO-9.

Water diffusion on solid surfaces is limited, so wettability is often improved by adding surfactants. Surfactants reduce surface tension, allowing liquids to spread more evenly over solid surfaces [28]. Figure 2 illustrates that as the SDBS content increased, the surface wettability transitioned from lipophilic to hydrophilic. The results show that the SDBS-AEOn composite system significantly reduced the contact angle and improved the wetting properties of lipophilic surfaces. Although SDBS greatly lowered the contact angle, the reduction did not continue uniformly with increasing SDBS, likely due to the limitation imposed by the benzene ring structure on hydrocarbon chain extension at the solid surface. The contact angle of the SDBS-AEO-9 mixture on a hydrophobic surface was similar to that of TX-100 when X_SDBS_ = 0.1, indicating these two surfactants have comparable surface activity characteristics. Similar results were also shown in the surface tension curve.

#### 3.3.2. Foam Performance

**Figure 3:**
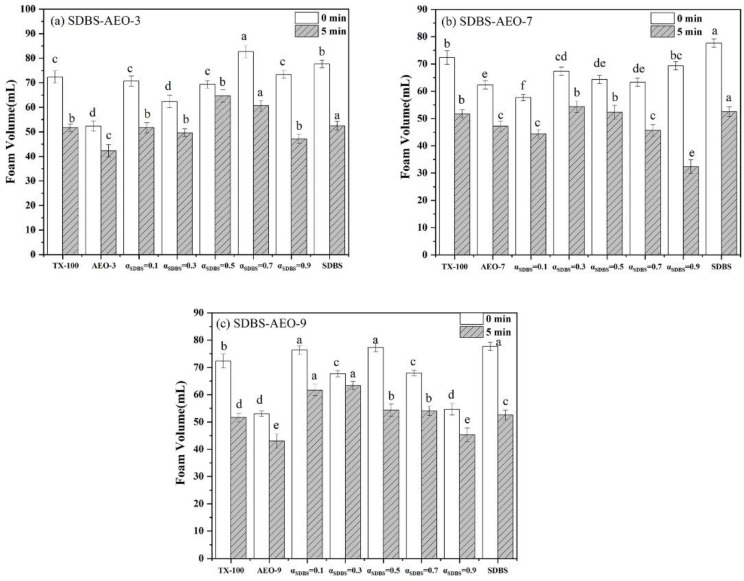
Foaming and stabilizing properties of SDBS-AEOn (0.5 g·L^−1^) systems. (**a**) SDBS-AEO-3, (**b**) SDBS-AEO-7, (**c**) SDBS-AEO-9. Different letters on the column represent significant levels of difference.

Figure 3 illustrates the foaming capacity and foam stability of the SDBS-AEOn mixed surfactant system. Among the surfactants with different EO numbers (*n* = 3, 6, 9), the foam performance of AEO-9 was higher than that of the other surfactants. As the number of the EO increases, the intermolecular hydrogen bonding increases, which may increase the molecular interaction and enhance the foaming ability. The non-ionic surfactant AEO and the anionic surfactant SDBS have good foaming properties when combined. In a mixed surfactant system, all mixed surfactant systems have higher foaming and foam stability than individual systems due to the synergistic effect. The CMC of a surfactant can be a good indicator of its efficiency as a foaming agent, especially in the SDBS-AEO-9 system. It is found that the lower the CMC of the mixed surfactant, the higher the efficiency of the surfactant as a foaming agent and the higher the stability of the foam. The mixed surfactant SDBS-AEO-9 (1:9) exhibited good foam stability, which was due to the high synergistic effect between SDBS and AEO-9 at this ratio. SDBS-AEO-9 (1:9) has the foam performance closest to TX-100, which is expected to provide a solution for the displacing of TX-100 in terms of a foaming agent.

#### 3.3.3. Emulsification Performance

**Figure 4:**
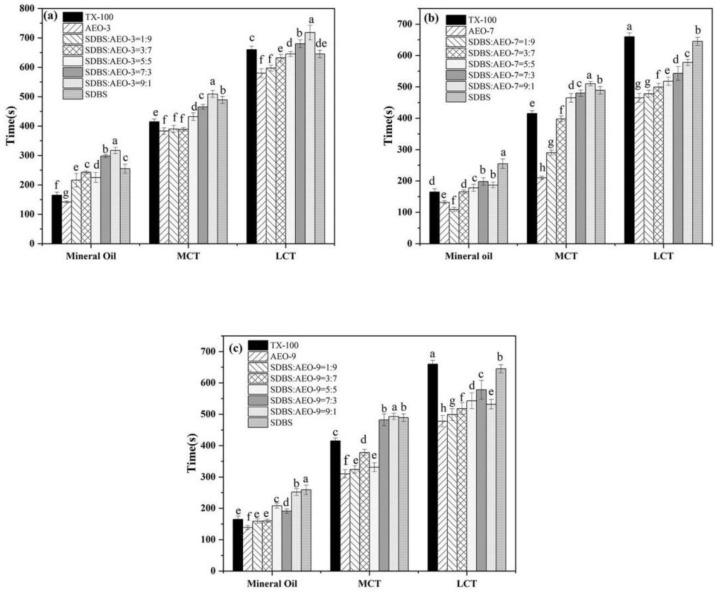
Emulsification properties of SDBS-AEOn mixture (1 g·L^−1^) mixture. (**a**) SDBS-AEO-3, (**b**) SDBS-AEO-7, (**c**) SDBS-AEO-9. Different letters on the column represent significant levels of difference.

Emulsification involves mixing two immiscible liquids by adding emulsifiers to create a stable liquid–liquid dispersion system. Surfactants, acting as emulsifiers, stabilize oil/water emulsions by reducing interfacial tension and coating droplets to prevent collisions [29]. The effectiveness of the emulsification was assessed by the demulsification time: longer separation times indicate a stronger emulsification ability. Figure 4 illustrates the emulsification capacity of the SDBS-AEOn mixture at different ratios. In the process of emulsification, the emulsification showed an upward trend with the growth of the EO chain, and the emulsification was the strongest when the EO number was 9. SDBS and AEO-9 can form intramolecular hydrogen bonds in aqueous solutions, which is conducive to the formation of stable liquid oil/water films. The ability of the mixed surfactants to emulsify liquid paraffin was significantly better than that of monomeric surfactants, which may be due to the fact that the combination of surfactants was more conducive to encapsulating oil-soluble components. The mixed surfactant system SDBS-AEO-9 has a good emulsifying ability for an LCT/water system. It is worth mentioning that SDBS-AEO-9 (1:9) has the closest emulsion stability to TX-100 for liquid paraffin, so it has the potential to replace TX-100 in emulsifying and dispersing systems.

#### 3.3.4. Hemolytic Activity

**Figure 5:**
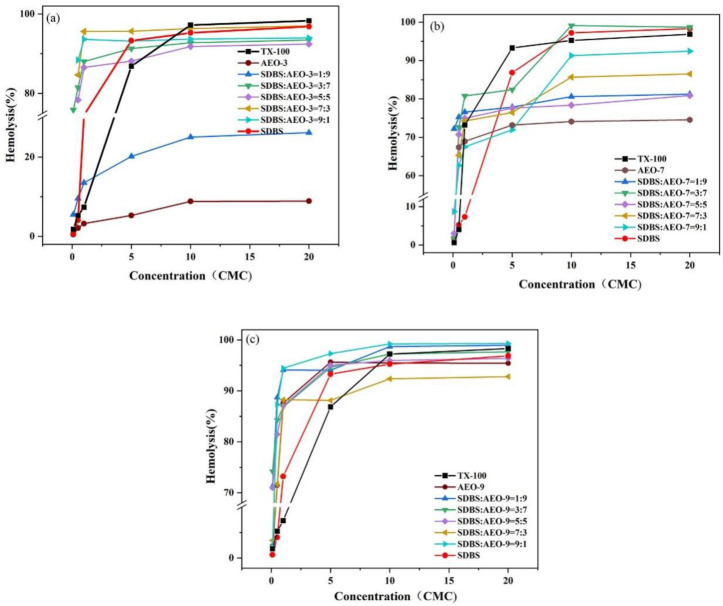
Hemolytic activity of SDBS-AEOn complex system (0.1~20 CMC). (**a**) SDBS-AEO-3, (**b**) SDBS-AEO-7, (**c**) SDBS-AEO-9.

Surfactants that adhere to cell membranes can cause hemolysis, convert liposomes into micelles, and disrupt the membrane [30,31]. TX-100 can dissolve cell membrane lipids and enhanced membrane permeability, making it widely used in biological detection [32,33]. As shown in Figure 5, the hemolytic activity of the SDBS-AEOn complex system across concentrations ranging from 0.1 to 20 CMC is illustrated. The results indicate that the relative hemolytic activity of AEO depended on its EO number: the higher the EO number, the higher the degree of hemolysis. For example, the hemolysis rates of AEO-3 and AEO-9 were 8.3% and 92% at 20 CMC concentrations, respectively. One possible explanation is that the increased hydrophilicity enhanced the permeability of surfactant molecules to cell membranes, leading to the rupture of red blood cells. Between 5 CMC and 20 CMC, the hemolysis curves for SDBS-AEO-7 (1:9) and SDBS-AEO-9 (1:9) overlapped with that of TX-100, indicating that the SDBS-AEO combination could effectively substitute for TX-100.

### 3.4. Replacement of TX-100 by SDBS-AEO Mixture in Sensor Making

**Figure 6:**
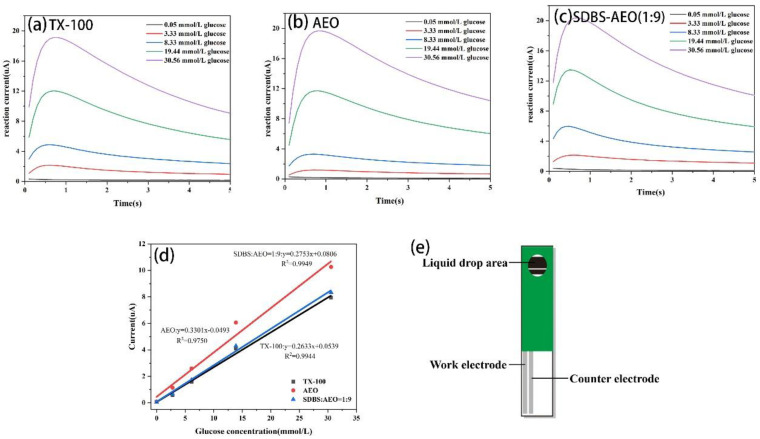
Reaction current curves of biosensor test strips prepared with different surfactants. (**a**) TX-100. (**b**) AEO. (**c**) SDBS: AEO = 1:9. (**d**) Linear regression analysis of electrochemical test results. (**e**) Schematic diagram of the biosensor test strip.

The above results show that SDBS-AEO-9 (1:9) has a similar interfacial activity to TX-100. As an initial application attempt, we evaluated the feasibility of SDBS-AEO as a substitute for TX-100 in the production of biosensors. Figure 6a–c display the response curves of a blood glucose biosensor in the presence of various surfactants. The biosensor test strips containing TX-100 and SDBS-AEO-9 (1:9) showed a significant increase in reaction signal with a good linear gradient. As shown in Figure 6d, a linear regression analysis was performed using the average current of each glucose concentration at five seconds. The results of the linear test show that AEO had the highest regression slope at 0.3301 but a lower correlation coefficient (R^2^). On the other hand, TX-100 and SDBS-AEO (1:9) had a correlation value close to 1, suggesting their optimal linearity performance. With the highest R^2^ value and the reaction signal linear regression slope that is closest to TX-100, SDBS-AEO-9 (1:9) is the most appropriate replacement for TX-100. Overall, it is expected that SDBS-AEO (1:9) will exhibit comparable properties to TX-100, resulting in similar signal transmission effects during biological detection.

## 4. Conclusions

The purpose of this study was to find an alternative surfactant to TX-100, which has been widely used in sensor manufacturing. The ionic surfactant SDBS and the series of non-ionic surfactants AEOn with a different hydrophilicity were selected for formulating alternatives to TX100. The amount of EO and the ratio of the two surfactants were screened. It was found that interfacial tension and permeation properties increase with an increasing EO number. The SDBS-AEO-9 (1:9) binary system was found to exhibit similar interfacial physicochemical properties, such as surface activity, dynamic interfacial activity, critical micelle concentration, and interfacial adsorption. The macroscopic performance of TX-100 in terms of wetting, foaming, emulsification, and solubilizing ability were also matched by the properties of SDBS-AEO-9 (1:9). In addition, the possibility of SDBS-AEO replacing TX-100 was confirmed by the production of glucose sensors. This article provides theoretical and experimental methods for the systematic screening of TX-100 alternatives for biosensors. It also provides important information on the interfacial activity mechanism of the amphiphilic components in the sensor.

## Figures and Tables

**Table 1:** Basic surface properties * of surfactant monomer solutions.

Category	Surfactants	CMC (mmol/L)	*γ_CMC_* (mN/m)	*pC*_20_	*Γ_max_* (μmol/m^2^)	*A_min_* (nm^2^)
	TX-100	0.28	29.81	4.69	3.39	0.48
Anionic	SDBS	0.25	31.81	4.34	4.68	0.35
	SLS	7.07	33.26	3.76	2.39	0.69
	NLSS	3.71	41.17	3.45	1.85	0.89
	SDS	7.41	44.78	2.94	1.48	1.11
	SBS	10.20	33.27	3.79	2.52	0.65
	AES	0.50	35.40	3.84	2.84	0.58
Non-ionic	AEO	0.16	33.81	4.81	3.65	0.45
	Brij58	0.033	43.21	3.77	2.88	0.57
	TW-80	0.012	40.48	3.48	2.47	0.67

* *CMC* is critical micelle concentration. *γ_CMC_* is surface tension at critical micelle concentration. *pC*_20_ is negative logarithm of surfactant concentration required to reduce surface tension by 20 mN/m, *Γ_max_* is maximum saturated adsorption capacity, and *A_min_* is minimum molecular cross-sectional area.

**Table 2:** Synergistic parameters * of the SDBS-AEOn complex system.

Surfactant	*α*_1_	*CMC* (mmol/L)	*γ_CMC_* (mN/m)	*X_1_*	*β^M^*	$\ln\left|\frac{{CMC}_{1}}{{CMC}_{2}}\right|$	$X_{1}^{s}$	$\beta^{s}$	$\ln\left|\frac{c_{1}^{0}}{c_{2}^{0}}\right|$	${\Delta H}^{M}$ (KJ/mol)	${\Delta G}^{M}$ (KJ/mol)	${\Delta S}^{M}$ (J/mol)
AEO-3	0.1	0.047	28.23	0.07	−1.52	1.43	0.19	−1.20	1.20	−1.58	−3.944	7.93
	0.3	0.059	29.58	0.19	−1.51		0.24	−1.33		−0.91	−2.262	4.52
	0.5	0.087	30.28	0.35	−1.66		0.31	−1.10		−0.29	−1.683	4.66
	0.7	0.112	29.20	0.49	−1.73		0.44	−1.25		−0.34	−1.989	5.55
	0.9	0.162	28.41	0.71	−2.39		0.67	−1.86		−0.41	−2.033	5.44
AEO-7	0.1	0.112	30.88	0.12	−1.28	0.85	0.09	−0.64	0.66	−0.21	−0.580	1.24
	0.3	0.120	33.07	0.19	−0.43		0.21	−0.67		−0.28	−1.438	3.87
	0.5	0.132	30.95	0.37	−0.86		0.39	−0.81		−0.60	−1.924	4.43
	0.7	0.166	31.04	0.50	−0.31		0.53	−0.55		−0.76	−1.905	3.85
	0.9	0.195	29.62	0.76	−1.45		0.72	−1.20		−0.36	−1.774	4.74
AEO-9	0.1	0.140	30.01	0.11	−1.14	0.39	0.18	−1.56	0.51	−0.92	−2.651	5.79
	0.3	0.170	33.42	0.26	−0.45		0.35	−0.62		−0.56	−1.983	4.77
	0.5	0.190	32.35	0.43	−0.39		0.51	−1.24		−0.21	−1.887	5.61
	0.7	0.210	31.51	0.65	−0.85		0.71	−0.71		−0.25	−1.921	5.60
	0.9	0.230	31.18	0.78	−0.98		0.75	−1.32		−1.12	−2.352	4.15

* *X*_1_ is the molar fraction of component SDBS in mixed micelles, *β^M^* is the interaction parameters in micelles, ${\;X}_{1}^{s}$ is the molar fraction of SDBS in the mixed monolayer, $\beta^{s}$ is the interaction parameters in hybrid monolayers. ${\Delta H}^{M}$ is the mixed enthalpy change,${\;\Delta G}^{M}\;$ is the mixed free energy change, ${\;\Delta S}^{M}$ is the mixed entropy change.

**Table 3:** Dynamic adsorption parameters * of SDBS-AEOn complex system.

Surfactant	$\alpha_{SDBS}$	C < CMC				C > CMC		
		Initial Adsorption		Late Adsorption		${\left(\frac{d\gamma }{{dt}^{-1/2}}\right)}_{cmc}$	$\frac{d\gamma }{{dt}^{-1}}$	*K* (s^−1^)
		$\frac{d\gamma }{{dt}^{1/2}}$ (mN/m·s^1/2^)	Da (m^2^/s)	$\frac{d\gamma }{{dt}^{-1/2}}$ (mN/m·s^1/2^)	Da (m^2^/s)			
AEO-3	0	−2.14	0.08	11.83	0.04	13.45	7.78	3.81
	0.1	−4.24	0.26	14.66	0.11	19.73	15.58	2.04
	0.3	−5.95	0.16	14.01	0.12	17.80	16.01	1.57
	0.5	−4.13	0.15	8.07	0.06	25.67	21.87	1.76
	0.7	−4.65	0.27	9.21	0.06	21.43	19.08	1.61
	0.9	−6.68	0.44	11.19	0.11	24.67	19.46	2.05
	1	−3.53	0.30	13.99	0.03	13.90	2.70	33.76
AEO-7	0	−5.84	1.52	20.18	0.70	23.10	15.61	2.79
	0.1	−7.96	4.49	17.36	0.65	21.45	17.98	1.81
	0.3	−6.31	1.50	17.63	0.35	15.98	11.32	2.54
	0.5	−7.71	1.54	18.48	0.44	12.34	9.98	1.95
	0.7	−7.00	0.61	19.30	0.23	9.95	5.89	3.64
	0.9	−6.19	0.54	17.94	0.13	13.56	9.58	2.55
	1	−3.53	0.30	13.99	0.03	13.90	2.70	33.76
AEO-9	0	−4.77	3.17	43.58	2.08	12.54	9.45	2.24
	0.1	−12.7	5.93	40.11	1.03	9.54	9.20	1.31
	0.3	−8.97	0.39	29.02	0.3	11.54	9.07	2.06
	0.5	−8.71	3.63	27.95	2.01	6.09	4.65	2.19
	0.7	−5.73	1.81	27.02	0.33	7.65	6.70	1.66
	0.9	−8.31	3.98	52.8	1.92	10.98	8.96	1.91
	1	−3.53	0.30	13.99	0.03	13.90	2.70	33.76

* $D_{a}$ is the apparent diffusion coefficient, and *k* is the micelle dissociation rate constant.

## Data Availability

The data presented in this study are available on request from the corresponding author.
